# The Evaluation of Immune Checkpoint Inhibitors and BRAF/MEK Inhibitors in Different Therapy Lines for Metastatic Melanoma: A Retrospective Study

**DOI:** 10.3390/jcm13185560

**Published:** 2024-09-19

**Authors:** Saki Okuda-Hiwatashi, Ryo Amagai, Taku Fujimura, Yumi Kambayashi, Manami Watanabe-Takahashi, Emi Yamazaki, Erika Tamabuchi, Chisato Itabashi, Akira Hashimoto, Yoshihide Asano

**Affiliations:** Department of Dermatology, Tohoku University Graduate School of Medicine, Sendai 980-8574, Japan; saki.0127@derma.med.tohoku.ac.jp (S.O.-H.); amagai@derma.med.tohoku.ac.jp (R.A.); yumi1001@hosp.tohoku.ac.jp (Y.K.); manami777@derma.med.tohoku.ac.jp (M.W.-T.); hemiura0804@derma.med.tohoku.ac.jp (E.Y.); eri-tama@derma.med.tohoku.ac.jp (E.T.); itabashichisato8@derma.med.tohoku.ac.jp (C.I.); hashi-a@derma.med.tohoku.ac.jp (A.H.); yasano@derma.med.tohoku.ac.jp (Y.A.)

**Keywords:** melanoma, nivolumab plus ipilimumab, BRAFi/MEKi, therapy sequence, efficacy, adverse events

## Abstract

**Background**: Nivolumab plus ipilimumab (nivo/ipi) combination therapy is highly effective in treating advanced melanoma, but serious immune-related adverse events (irAEs) are prevalent. The overall response rate (ORR) of the BRAF inhibitor plus MEK inhibitor (BRAFi/MEKi) combination therapy for *BRAF^V600^*-mutant advanced melanoma surpasses that of immune checkpoint inhibitors (ICIs). However, the OS and PFS of BRAFi/MEKi combination therapy are inferior to those of ICIs. **Methods**: We retrospectively evaluated 22 melanoma patients treated with nivo/ipi therapy and 13 patients treated with encorafenib plus binimetinib (enco/bini) between November 2018 and July 2023. **Results**: The ORR of nivo/ipi for metastatic melanoma patients was significantly higher in the first-line cohort [60.0% (95% CI: 31.2–83.3%)] than in the second-line or beyond cohort [8.3% (95% CI: 0–37.5%)], whereas the ORR of enco/bini was comparable between the first-line cohort [75.0% (95% CI: 28.9–96.6%)] and the second-line or beyond cohort [77.8% (95% CI: 44.3–94.7%)]. The median PFS of nivo/ipi significantly improved in the first-line cohort [7.7 months (95% CI: 2.0–11.9)] compared to the second-line or beyond cohort [2.3 months (95% CI: 0.5–6.0)] (*p* = 0.0109). In addition to efficacy, the incidence of grade 3 or greater AEs was comparable in the first-line and second-line or beyond cohorts. **Conclusions**: Although our present data are based on a small number of cases, they suggest that nivo/ipi should be administered as the first-line therapy for the treatment of *BRAF^V600^*-mutant metastatic melanoma, rather than enco/bini, aligning with findings from previous clinical trials.

## 1. Introduction

Nivolumab plus ipilimumab (nivo/ipi) combination therapy is highly effective in treating advanced melanoma, but serious immune-related adverse events (irAEs) are prevalent [1,2]. Indeed, a previous report suggests that the overall response rate (ORR) to nivo/ipi combination therapy is higher than that of nivolumab monotherapy [57.6% (95% CI: 52.0–63.2%) vs. 43.7% (95% CI: 38.1–49.3%)] [1]. Moreover, a 5-year overall survival (OS) rate is significantly better with nivo/ipi combination therapy (38%) than with nivolumab monotherapy (28%) among patients with a high tumor volume and elevated LDH levels, suggesting that nivo/ipi may be effective in patients with later stage melanoma [2]. In addition, the 5-year progression-free survival (PFS) rate is also improved with nivo/ipi combination therapy in patients with high-volume disease and elevated LDH levels [2]. Notably, a subgroup analysis of the CheckMate 067 trial demonstrated the benefit of nivo/ipi combination therapy for the *BRAF*-mutated advanced melanoma group [1]. In fact, the 5-year OS rate in patients with *BRAF*-mutated tumors significantly improved in the nivo/ipi combination group (60%) compared to the nivolumab monotherapy group (46%) [2]. In addition, the 5-year PFS rate also improved with nivo/ipi combination therapy (38%) compared to nivolumab monotherapy (22%) in patients with tumors harboring BRAF mutations. As the efficacy of BRAF inhibitor plus MEK inhibitor (BRAFi/MEKi) is limited in patients with elevated LDH levels [3,4], nivo/ipi combination therapy could be a first-line immunotherapy for BRAF-mutant advanced melanoma with a high tumor burden. In fact, several clinical trials have provided data to recommend that ipi/nivo should be given first [5,6,7]. On the other hand, the efficacy of immune checkpoint inhibitors (ICIs) in the Japanese cohort is lower than in Caucasians [8,9,10]. Furthermore, nivo/ipi combination therapy is less effective in cases of anti-PD-1 antibody-refractory melanoma [11,12], though a recent exploratory clinical trial has suggested useful combination drugs for anti-PD-1 antibody-resistant unresectable melanoma [13]. Therefore, the administration of nivo/ipi combination therapy necessitates a case-by-case determination in the real world, particularly in the Japanese context.

On the other hand, the ORR of BRAFi/MEKi combination therapy for *BRAFV600*-mutant advanced melanoma surpasses that of other anti-melanoma drugs, including ICIs [1,3,14]. Among them, encorafenib plus binimetinib (enco/bini) combination therapy is one of the optimal chemotherapies for *BRAF*-mutated advanced melanoma [14,15]. The ORR to enco/bini combination therapy (as calculated by a masked independent central review) was 64% [14]. The enco/bini combination therapy significantly improved PFS compared to an encorafenib monotherapy group [hazard ratio (HR) 0.77, 95% CI: 0.59–1.00; *p* = 0.05] [14]. Moreover, the median OS for enco/bini vs. encorafenib vs. vemurafenib was 33.6 months (95% CI: 24.4–39.2 months) vs. 23.5 months (95% CI: 19.6–33.6 months) vs. 16.9 months (95% CI: 14.0–24.5 months), respectively [14], suggesting that the BRAFi/MEKi combination therapy is superior to the BRAFi monotherapy. However, the 5-year overall survival (OS) rate and progression-free survival (PFS) rate of BRAFi/MEKi combination therapy are inferior to those of ICIs [3,14]. Notably, in contrast to ICIs for unresectable melanoma, the efficacy and safety profiles of BRAF/iMEKi combination therapy in Japan are comparable to those in Caucasian countries [3,14,16,17,18]. Consequently, the optimal treatment sequence for *BRAF^V600^*-mutant advanced melanoma remains a matter of controversy [13,14,15]. The objective of this study is to evaluate these two chemotherapy regimens in a real-world setting, aiming to optimize each regimen.

## 2. Materials and Methods

We retrospectively reviewed a database collected by the Department of Dermatology, Tohoku University Hospital, and identified 22 patients with melanoma treated with nivolumab plus ipilimumab (nivo/ipi) and 13 patients treated with encorafenib plus binimetinib (enco/bini) between November 2018 and July 2023. The protocol was approved by the ethics committee of the Tohoku University Graduate School of Medicine, Sendai, Japan (2021-1-1213). Data such as the male-to-female ratio, age, Clark’s histological classification, Bastian’s molecular-genetic classification, clinical stage, systemic therapy line, number of metastatic tumors, LDH level and BRAF states were collected.

### 2.1. Outcome Measures

The primary endpoint of this study was the response rate based on the criteria of the Response Evaluation Criteria in Solid Tumours (RECIST) version 1.1. Secondary outcome measures included progression-free survival (PFS), overall survival (OS) and adverse events (AEs) graded using the National Cancer Institute Common Terminology Criteria for Adverse Events, version 4.0. For this study, PFS was defined as the time from treatment initiation to disease progression or death from any cause, and OS was defined as the time from the initiation of treatment in the clinical trial to death from any cause. All analyses were based on investigator assessment.

### 2.2. Safety Assessment

The safety assessment included the collection of data on AEs, results of clinical laboratory tests and physical examinations and vital signs. The severity grade (Common Terminology Criteria for Adverse Events, version 4.0—Japan Clinical Oncology Group) and the relationship to each therapy protocol were determined for each AE.

### 2.3. Statistical Analysis

The PFS and OS rates were assessed for each group using the Kaplan–Meier method. The log-rank test was employed to compare survival between groups. Hazard ratios (HRs) and 95% confidence intervals (95% CIs) were determined using Cox’s proportional hazards model in univariate analysis. The significance level for the log-rank test was set at a two-sided α of 0.05. All statistical analyses were performed using EZR (Saitama Medical Center, Saitama, Japan), a graphical user interface for R (The R Foundation for Statistical Computing, Vienna, Austria). Specifically, it is a modified version of the R commander designed to incorporate statistical functions commonly used in biostatistics.

## 3. Results

### 3.1. Demographic Data

Patient demographics for the nivo/ipi cohort are shown in Table 1. The melanoma subtypes included four cases of superficial spreading melanoma (SSM) (18%), seven cases of nodular melanoma (NM) (32%), five cases of acral lentiginous melanoma (ALM) (23%) and six cases of mucosal melanoma (27%) based on Clark’s histological classification. According to Bastian’s classification, three cases had high cumulative sun damage (CSD) (13%), eight cases had low CSD (36%), five cases had acral melanoma (23%) and six cases had non-cutaneous melanoma (mucosal melanoma). Ten cases were treated with nivo/ipi as the first-line therapy (45%), and twelve cases received it as the second-line therapy or beyond (55%). Serum LDH levels were within the normal range in fifteen cases (68%) and high in seven cases (32%). Fifteen cases had *BRAF* wild-type melanoma (68%), six had *BRAF^V600E^*-mutated melanoma (27%) and one case was not evaluated (5%). Among patients with *BRAF^V600E^*-mutated melanoma, 2 patients were in the first-line cohort, and 4 patients were in the second-line or beyond cohort. In other words, 20% of patients in the first-line cohort had *BRAF^V600E^*-mutated melanoma, whereas 33% of patients in the second-line or beyond cohort had *BRAF^V600E^*-mutated melanoma.

Patient demographics for the enco/bini cohort are shown in Table 1. The melanoma subtypes included two cases of SSM (15%), six cases of NM (46%), three cases of ALM (23%) and two cases of melanoma of unknown origin (15%) based on Clark’s histological classification. According to Bastian’s classification, nine cases had low CSD (69%), two cases had acral melanoma (15%) and two cases had melanoma of unknown origin. Four cases were treated with the enco/bini regimen as the first-line (31%), and eleven cases received it as the second-line or beyond (69%). Serum LDH levels were within the normal range in nine cases (69%) and high in four cases (31%). Twelve cases had *BRAF^V600E^*-mutated melanoma (93%), and one case had *BRAF^V600K^*-mutated melanoma (7%).

### 3.2. Efficacy

Among the 22 patients treated with nivo/ipi, the response rate was 31.8% (95% CI: 16.2–52.9%), including 1 case of complete response (CR) (4.5%), 6 cases of partial response (PR) (27.2%), 6 cases of stable disease (SD) (27.2%) and 9 cases of progressive disease (PD) (40.9%), and the disease control rate (DCR) was 59.1% (95% CI: 38.7–76.8%). In the subgroup of 10 patients treated with nivo/ipi as the first-line cohort, the response rate was 60.0% (95% CI: 31.2–83.3%), including 1 case of CR (10.0%), 5 cases of PR (50.0%), 3 cases of SD (30.0%) and 1 case of PD (10.0%), and the DCR was 90.0% (95% CI: 57.4–100.4%). Among the 12 patients treated with nivo/ipi as the second-line or beyond cohort, the response rate was 8.3% (95% CI: 0–37.5%), including 1 case of PR (8.3%), 3 cases of SD (25.0%) and 8 cases of PD (66.7%), and the DCR was 33.3% (95% CI: 13.6–61.2%) (Supplementary Tables S1a and S2).

Of the 13 patients treated with enco/bini, the efficacy was 76.9% (95% CI: 49.0–92.5%), including 4 cases of CR (30.8%), 6 cases of PR (46.2%), 2 cases of SD (15.4%) and 1 case of PD (7.7%), and the DCR was 92.3% (95% CI: 64.6–100.7%). Among the four patients treated with enco/bini as the first-line cohort, the efficacy was 75.0% (95% CI: 28.9–96.6%), including one case of CR (25.0%), two cases of PR (50.0%) and one case of PD (25.0%), and the DCR was 75.0% (95% CI: 28.9–96.6%). Among the nine patients treated with enco/bini as the second-line or beyond, the efficacy was 77.8% (95% CI: 44.3–94.7%), including three cases of CR (33.3%), four cases of PR (44.4%) and two cases of SD (22.2%), and the DCR was 77.8% (95% CI: 44.3–94.7%) (Supplementary Tables S1b and S2).

**Figure 1:**
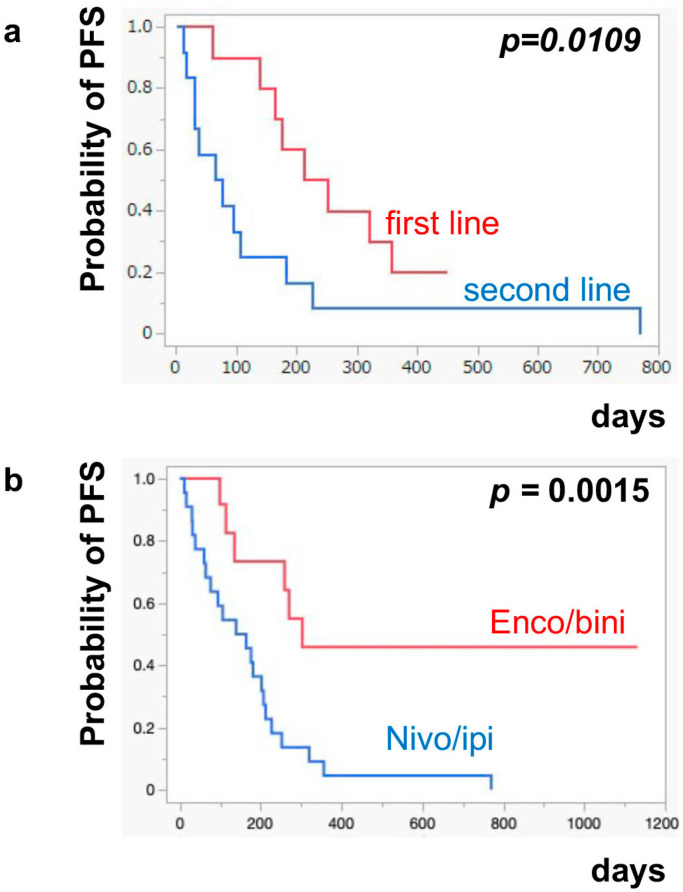

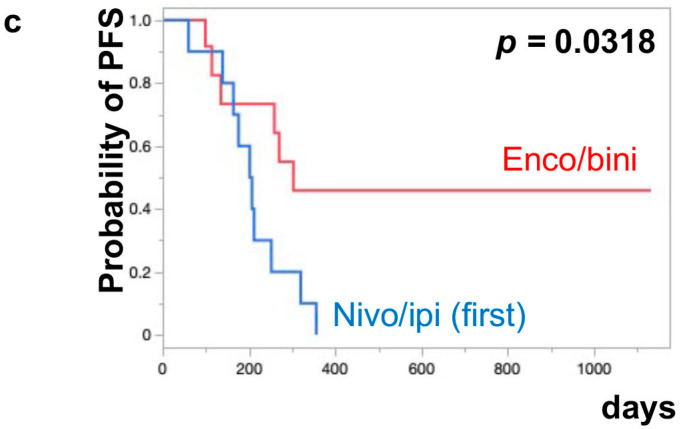
The median PFS of (**a**) nivo/ipi in the first-line cohort vs. the second-line or beyond cohort, (**b**) the enco/bini cohort vs. the nivo/ipi cohort (**c**) and the enco/bini cohort vs. nivo/ipi in the first-line cohort.

**Figure 2:**
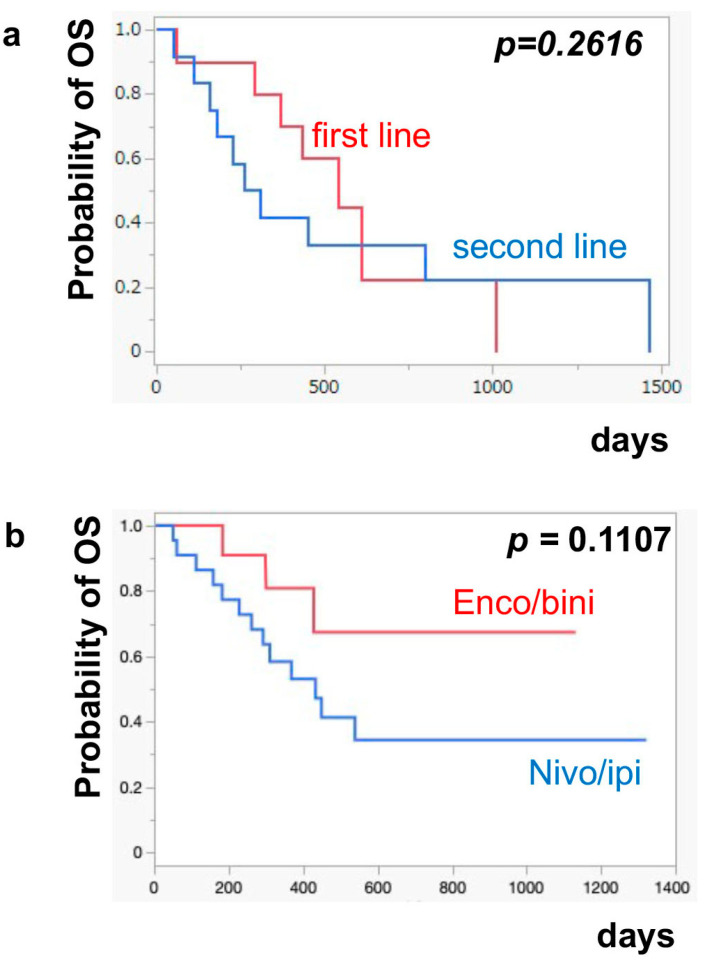

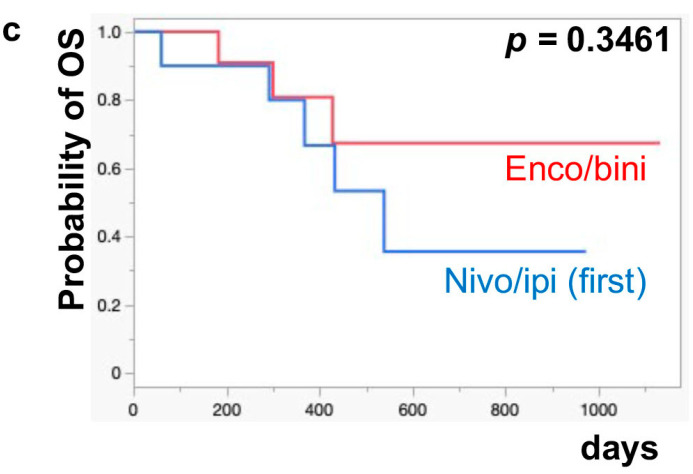
The median OS of (**a**) nivo/ipi in the first-line cohort vs. the second-line or beyond cohort, (**b**) the enco/bini cohort vs. the nivo/ipi cohort (**c**) and the enco/bini cohort vs. nivo/ipi in the first-line cohort.

The median PFS of nivo/ipi was significantly improved in the first-line cohort [7.7 months (95% CI: 2.0–11.9)] compared to the second-line or beyond cohort [2.3 months (95% CI: 0.5–6.0)] (*p* = 0.0109) (Figure 1a). However, there was no significant difference in the PFS of the enco/bini between the first-line cohort and the second-line or beyond cohort (neither cohort reached the median). The PFS was significantly improved in the enco/bini cohort compared to the nivo/ipi cohort (*p* = 0.0015) (Figure 1b) and even improved compared to the ipi/nivo first-line cohort (*p* = 0.0318) (Figure 1c). There was no significant difference in PFS between the enco/bini first-line cohort and the ipi/nivo first-line cohort (*p* = 0.2886). In BRAF-mutant melanoma, there was no significant difference in the PFS between the enco/bini first-line cohort and the ipi/nivo first-line cohort (*p* = 0.6104). The median OS of the nivo/ipi first-line and second-line cohort was 18.0 months (95% CI: 2.0–33.6) and 9.5 months (95% CI: 3.7–26.7) (*p* = 0.2616), respectively. The median OS of the enco/bini first-line and second-line cohorts was not reached. There was no significant difference in the OS between the nivo/ipi first-line and second-line cohort (*p* = 0.2616). There was no significant difference in the OS between the enco/bini cohort and the ipi/nivo cohort (*p* = 0.1107) (Figure 2b,c), or the enco/bini cohort and the ipi/nivo first-line cohort (*p* = 0.3461).

### 3.3. Safety Profile

Eighteen cases of grade 3 or higher AEs with nivo/ipi were reported in 12 patients [54.5% (95% CI: 34.6–73.1%)], including 2 cases of liver dysfunction (20%) in the first-line cohort and 4 cases in the second-line or beyond cohort. Further details of grade 3 or higher AEs with nivo/ipi in each cohort are described in Table 2a. Four cases of grade 3 or higher AEs with nivo/ipi were reported in 4 patients [30.8% (95% CI: 12.4–58.0%)], including 2 cases of liver dysfunction (15%), 1 case of fever (8%) and 1 case of uveitis (8%) (Table 2b).

## 4. Discussion

There has been considerable debate regarding the sequential use of nivo/ipi combination therapy and *BRAF/MEKi* combination therapy. Recent clinical trials suggest a preference for nivo/ipi combination therapy as a first-line regimen in Caucasian cohorts [5,19,20]. For instance, the DREAMseq trial indicates a 2-year OS of 71.8% (95% CI: 62.5–79.1) for the nivo/ipi followed by the dabrafenib plus trametinib (dab/tra) cohort and 51.5% (95% CI: 41.7–60.4; log-rank *p* = 0.010) for the dab/tra followed by the nivo/ipi cohort in the treatment of *BRAFV600*-mutant metastatic melanoma [19]. Another clinical trial, the SECOMBIT trial, also demonstrates a superior OS in the nivo/ipi prior cohort compared to the enco/bini prior cohort [5,20]. These clinical trials suggest that nivo/ipi should be administered before *BRAF/MEKi* in *BRAFV600*-mutant metastatic melanoma. Notably, about 40% of Japanese melanomas have acral melanoma [21], which possesses a low tumor mutation burden [22], which makes ICI less effective in treating them. Moreover, not only in the Japanese population but also in East Asian, Hispanic and African patients, the efficacy of ICI is lower than that in Caucasian patients [10,23]. Furthermore, several clinical trials have provided data to recommend that ipi/nivo be given first [5,6,7]. Moreover, previous reports also suggested that the efficacies of nivo/ipi in second-line or beyond are much lower compared to first-line therapy [24,25]. Given the lower efficacy of ICIs in the Japanese cohort compared to Caucasians [8,9] and the reduced effectiveness of nivo/ipi combination therapy in cases of anti-PD-1 antibody-refractory melanoma [11,12], assessing the efficacy of nivo/ipi combination therapy based on treatment lines is crucial for determining the sequence of anti-melanoma therapy, especially for *BRAFV600*-mutant metastatic melanoma [5,6,26].

In our present study, the efficacy of nivo/ipi for metastatic melanoma patients was significantly higher in the first-line cohort [60.0% (95% CI: 31.2–83.3%)] compared to the second-line or beyond cohort [8.3% (95% CI: 0–37.5%)]. Notably, 2 cases (20.0%) in the first-line setting were *BRAFV600E*-mutants, whereas 4 cases (33.3%) in the second-line or beyond setting were *BRAFV600E*-mutants. Although nivo/ipi is more effective for *BRAFV600*-mutant metastatic melanoma than *BRAFV600* wild-type melanoma 1, the first-line cohort exhibited higher efficacy than the second-line or beyond cohort. In addition, the median PFS of nivo/ipi was significantly prolonged in the first-line cohort [7.7 months (95% CI: 2.0–11.9)] compared to the second-line or beyond cohort [2.3 months (95% CI: 0.5–6.0)] (*p* = 0.0109). In addition to efficacy, the incidence of grade 3 or greater AEs is comparable in the first-line cohort and the second-line or beyond cohorts. These findings suggest that nivo/ipi should be considered as a first-line treatment, where appropriate, even for the treatment of *BRAFV600*-mutant metastatic melanoma.

In contrast to nivo/ipi, the ORR of enco/bini combination therapy in Japanese *BRAFV600*-mutant melanoma is comparable to that in Caucasian melanoma [16,17]. Indeed, in our present study, the efficacy of the 13 patients treated with enco/bini was 76.9% (95% CI: 49.0–92.5%), including high rates of CR (30.8%) and DCR (92.3%). Notably, the efficacy of enco/bini was comparable between the first-line cohort [75.0% (95% CI: 28.9–96.6%)] and the second-line or beyond cohort [77.8% (95% CI: 44.3–94.7%)]. In contrast to nivo/ipi, there was no significant difference in the PFS of enco/bini between the first-line cohort and the second-line or beyond cohort. These findings suggest that enco/bini may be applicable not only in the first-line setting but also in the second-line or beyond.

## 5. Conclusions

Taken together, despite being based on a small number of cases, our current data suggests that first-line therapy using nivo/ipi should be administered for the treatment of *BRAFV600*-mutant metastatic melanoma, consistent with previous clinical trials [5,19,20]. Since our present study was retrospective and performed on a limited number of patients, especially in the enco/bini cohort, and has no validation cohort, further samples will be needed to confirm these findings.

## Figures and Tables

**Table 1:** Patients treated with nivo/ipi: baseline characteristics *(n* = 22) and with enco/bini: baseline characteristics (*n* = 13).

	nivo/ipi	enco/bini
**Median Age**	68 (42–86)	57 (37–77)
**Sex**		
Male	14 (64%)	7 (54%)
Female	8 (36%)	6 (46%)
**Clark’s histological classification**		
Superficial spreading melanoma	4 (18%)	2 (15%)
Nodular melanoma	7 (32%)	6 (46%)
Acral lentiginous melanoma	5 (23%)	3 (23%)
Mucosal	6 (27%)	2 (15%)
**Acral, CSD, Non-CSD grouping**		
High CSD	3 (13%)	0 (0%)
Low CSD	8 (36%)	9 (69%)
Acral	5 (23%)	2 (15%)
Mucosal	6 (27%)	2 (15%)
**Clinical stage**		
Unresectable II	2 (9%)	0 (0%)
Unresectable III	1 (5%)	0 (0%)
IV	19 (86%)	13 (100%)
**Chemotherapy line**		
First-line	10 (45%)	4 (31%)
Second-line or beyond	12 (55%)	9 (69%)
**Number of metastatic tumors**		
0–2	14 (64%)	11 (85%)
3≤	8 (36%)	3 (15%)
**LDH level**		
Within normal range	15 (68%)	9 (69%)
High	7 (32%)	4 (31%)
**BRAF status**		
Wild type	15 (68%)	0 (0%)
Mutant	6 (27%)	12 (93%)
Not reported	1 (5%)	1 (7%)

**Table 2:** (a) Incident of grade 3 or more AEs in nivo/ipi cohort. (b) Incident of grade 3 or more AEs in enco/bini cohort.

(a)		
Grade 3 ≥ AEs	First-Line (*n* = 10)	Second-Line or Beyond (*n* = 12)
any	6 (60%)	6 (50%)
liver dysfunction	2 (20%)	4 (33%)
increased level of serum lipase	1 (10%)	1 (8%)
anemia	0	1 (8%)
encephalitis	1 (10%)	0
demyeling disease	1 (10%)	0
hypophysitis	1 (10%)	0
myasthenia gravis	1 (10%)	0
colitis	1 (10%)	1 (8%)
interstitial pneumonia	0	1 (8%)
nephritis	1 (10%)	0
skin rash	1 (10%)	0
**(b)**		
**Grade 3 ≥ AEs**	**First-Line (4)**	**Second-Line or Beyond (9)**
any	3 (75%)	1 (11%)
liver dysfunction	1 (25%)	1 (11%)
fever	1 (25%)	0
uveitis	1 (25%)	0

## Data Availability

The data that supports the findings of this study are available on request from the corresponding author, T.F. The data are not publicly available due to containing information that could compromise the privacy of research participants.

## Supplementary Materials

The following supporting information can be downloaded at: https://www.mdpi.com/article/10.3390/jcm13185560/s1, Supplementary Table S1. (a) The efficacy of nivo/ipi cohort. (b) The efficacy of enco/bini cohort. Supplementary Table S2. Stratified analysis of nivo/ipi cohort (first-line and second-line or beyond) and enco/bini cohort.
