# Supplementary material for: The Evaluation of Immune Checkpoint Inhibitors and BRAF/MEK Inhibitors in Different Therapy Lines for Metastatic Melanoma: A Retrospective Study
Source: J Clin Med. 2024 Sep 19;13(18):5560. doi: 10.3390/jcm13185560 (PMC11432506; doi:10.3390/jcm13185560)
Supplement: Supplementary file 1 [file jcm-13-05560-s001.zip › jcm-3169877-supplementary.pdf]

Supplemental Table S1

(a) The efficacy of nivo/ipi cohort

	All	First line	Second line or beyond
CR	1 (5%)	1 (10%)	0 (0%)
PR	6 (27%)	5 (50%)	1 (8%)
SD	6 (27%)	3 (30%)	3 (14%)
PD	9 (41%)	1 (10%)	8 (67%)

(b) The efficacy of enco/bini cohort

	All	First line	Second line or beyond
CR	4 (31%)	1 (25%)	3 (33%)
PR	6 (46%)	2 (50%)	4 (44%)
SD	2 (15%)	0 (0%)	2 (22%)
PD	1 (8%)	1 (25%)	0 (0%)

Supplemental Table S2. Stratified analysis of nivo/ipi cohort (first-line and second-line or beyond) and enco/bini cohort.

	first line	second line or beyond	enco/bini
Metastatic stage			
M1a	2	3	4
M1b	1	2	3
M1c	6	6	4
M1d	0	0	1
Unresectable	1	1	1
BRAF states			
BRAFB600E/K	2	2	13
BRAF L597	0	1	0
wild type	8	9	0
PFS			
M1a	402.5 (356 - 449)	16 (11 - 76)	103.5 (81 - 1039)
M1b	176	34.5 (31 - 38)	303 (45 - 319)
M1c	232 (60 - 437)	100 (30 - 770)	336.5 (135 - 1132)
M1d	-	-	259
Unresectable	139	181	474
OS			
M1a	528.5	112 (50 - 1223)	528.5 (81 - 1039)
M1b	1007	285.5 (261 - 31-)	319 (298 - 319)
M1c	400.5	338.5	351.5 (183 - 1132)
M1d	-	-	428
Unresectable	609	800	474
